# The association between day-to-day stress experiences, recovery, and work engagement among office workers in academia–An Ecological Momentary Assessment study

**DOI:** 10.1371/journal.pone.0281556

**Published:** 2023-02-21

**Authors:** Larissa Bolliger, Ellen Baele, Elena Colman, Gillian Debra, Junoš Lukan, Mitja Luštrek, Dirk De Bacquer, Els Clays

**Affiliations:** 1 Department of Public Health and Primary Care, Ghent University, Ghent, Belgium; 2 Department of Developmental, Personality, and Social Psychology, Ghent University, Ghent, Belgium; 3 Department of Intelligent Systems, Jožef Stefan Institute, Jožef Stefan International Postgraduate School, Ljubljana, Slovenia; Universidad de Zaragoza, SPAIN

## Abstract

**Objectives:**

This study aimed to investigate the associations between day-to-day work-related stress exposures (i.e., job demands and lack of job control), job strain, and next-day work engagement among office workers in academic settings. Additionally, we assessed the influence of psychological detachment and relaxation on next-day work engagement and tested for interaction effects of these recovery variables on the relationship between work-related stressors and next-day work engagement.

**Methods:**

Office workers from two academic settings in Belgium and Slovenia were recruited. This study is based on an Ecological Momentary Assessment (EMA) with a 15-working day data collection period using our self-developed STRAW smartphone application. Participants were asked repeatedly about their work-related stressors, work engagement, and recovery experiences. Fixed-effect model testing using random intercepts was applied to investigate within- and between-participant levels.

**Results:**

Our sample consisted of 55 participants and 2710 item measurements were analysed. A significant positive association was found between job control and next-day work engagement (β = 0.28, p < 0.001). Further, a significant negative association was found between job strain and next-day work engagement (β = −0.32, p = 0.05). Furthermore, relaxation was negatively associated with work engagement (β = −0.08, p = 0.03).

**Conclusions:**

This study confirmed previous results, such as higher job control being associated with higher work engagement and higher job strain predicting lower work engagement. An interesting result was the association of higher relaxation after the working day with a lower next-day work engagement. Further research investigating fluctuations in work-related stressors, work engagement, and recovery experiences is required.

## Introduction

Although working in academia was traditionally believed to be less stressful than most other professions [1], continuous changes in the sector throughout the past decades have caused increasing levels of psychological stress among academic staff [2–4]. This changing work environment in higher education brings up many concerns for academics [4, 5], such as an increasing trend of receiving only short-term contracts [4, 6] leading to increasing job insecurity [1, 5, 7], as well as a decrease in research funding opportunities [4, 5]. Rising stress levels in academia are due to high job demands such as long working hours [1], high workload [2, 5, 7, 8], and a large amount of administrative work [4, 5], with the latter two leading to experiencing time pressure [5]. Further stressors include limited freedom and independence in task organisation [1], poor leadership and organisational management [1, 5, 8], an increasing number of students and a lack of corresponding resources to provide the necessary teaching and support [3, 5], and insufficient recognition and rewards for the conducted work [2, 7].

The Job Demand-Control model by Karasek [9], one of the most influential occupational stress models, proposed that such high job demands can be balanced by an equivalent level of job control. Karasek suggested that a combination of high demands and high control are characteristics of active jobs, requiring high dedication and motivation for new learning opportunities [9]. Existing research from higher education confirmed Karasek’s work by showing that job resources, such as growth opportunities at work including autonomy, learning opportunities, task variety, and task significance, are leading to increased work engagement among academic staff [8, 10, 11]. As indicated by Schaufeli and Bakker [12], leading in research on work engagement, only 5% of the studies in occupational health psychology are focusing on such positive consequences of work environment exposures. Based on their concept, work engagement consists of a worker’s vigour, dedication, and absorption, making it a fulfilling and positive work-related state of mind [12]. According to previous research, highly engaged workers are about 78% more productive [13] and increased work engagement can lead to developing higher levels of resilience [14], self-efficacy, and optimism [15]. Recovery is another important part of the stress process, being the final step in the physiological stress responses of the body, aiming to stabilise itself and restore balance. This process is crucial since it decreases possible negative effects of stress on well-being [16]. Sonnentag and Fritz have conducted extensive research on mood regulation and work stress recovery [17], based on which they suggest that psychological detachment from work and relaxation [18, 19] are major strategies that people use to recover from job stressors [20]. Psychological detachment is the most researched activity to recover from work stress by mentally disconnecting from work during leisure time [20]. Evidence suggests that increased levels of psychological detachment decrease the risk of fatigue and burnout and increase physical and mental well-being [17, 21, 22]. Relaxation occurs by reducing either physical or mental activities or both. Existing literature has shown that relaxation is associated with increased vigour and decreased fatigue [21].

Previous research focused primarily on long-term exposures to psychosocial stress and their adverse impact on a multitude of health-related outcomes, primarily mental and cardiovascular diseases [23, 24]. Such chronic stress research has also shown the negative impact of job strain on work engagement [25, 26].

Contrary to such traditional research on chronic stress exposures and health-related outcomes, an Ecological Momentary Assessment (EMA) [27] allows the measurement of day-to-day work stressors and a multitude of stress outcomes, accounting for fluctuations and providing fine-grained insights. Based on Sonnentag [28], work engagement can fluctuate across time and situations. Sonnentag and Bayer [29] found that psychological detachment can change daily due to fluctuations in high time pressure, while van Hooff et al. [30] confirmed this fluctuating trend, describing that work-related recovery differed between employees as well as within employees over workdays and leisure time.

The purpose of this study using an intensive longitudinal design including an EMA for 15 consecutive workdays, was to investigate the relationships between self-perceived day-to-day job demands, job control, job strain, and next-day work engagement among office workers in academic settings. An additional aim was to assess the role of psychological detachment and relaxation, by researching whether these day-to-day recovery experiences relate to next-day work engagement or moderate the relationship between stress experiences and next-day work engagement.

## Materials and methods

The STROBE Statement [31], a checklist for observational studies, was used to report this study. This study is part of the STRAW project and more detailed information about it can be found in the protocol paper [32]. Although the STRAW project used a combination of different data collection methods, this paper focuses on EMA data only.

### Study setting, sample, and recruitment

The target sample included healthy office workers in academic settings with different occupations. Participants were not excluded based on mental or physical conditions. Job categories were divided into three groups: (1) administrative and technical staff (e.g., secretaries and IT support), (2) researchers without a PhD, and (3) researchers with a PhD. Participants were recruited via a variety of communication platforms from Ghent University in Belgium and the Jožef Stefan Institute in Slovenia, using the convenience sampling method. Such heterogeneity of office workers of different occupations in two different countries allows a better representation and increased external validity of the workforce in academic settings. The focus was set on researching associations between a variety of stress exposures and outcomes occurring in academic office work, without aiming to draw conclusions on the prevalence of stressors or stress consequences. To be eligible, participants had to meet the following criteria: (1) working at least 80% to be sufficiently exposed to a variety of different work stressors, (2) agreeing to install the STRAW smartphone application on their personal Android smartphone, (3) agreeing to continuously wear an Empatica wristband during waking hours of workdays, and (4) having oral permission from their supervisor to participate during work [32]. Recruitment of participants took place from October 2019 until June 2021.

### Study design and procedure

The STRAW project is based on an intensive longitudinal study design using an EMA, implemented in our self-developed STRAW smartphone application [33]. This EMA research method has several benefits: (1) it can be used to track experiences in real-world settings and in real-time using self-reports to capture daily experiences and contexts, (2) it allows multiple measurements per participant per day, (3) it enables data collection via digital platforms such as smartphone applications, (4) it allows for as little intrusion as possible, and (5) not only between-participant variations but also within-participant variations in everyday life experiences are taken into account [34].

The three-phased data collection procedure included: (1) an online baseline screening by means of a LimeSurvey questionnaire before day one and participant briefing on day one, (2) collection of EMA data for 15 consecutive workdays (weekends and days off excluded), and (3) participant debriefing on the last day of data collection. Data were collected from October 2020 until June 2021.

### EMA protocol

This study focused on several work-related stress exposures and stress outcomes, measured by means of EMAs including ten questionnaires in the overall STRAW project. The EMAs consisted of approximately 20 items during daytime and 40 items in the evening [32]. Since the used questionnaires were originally developed for chronic stress measurements, the items included in the EMAs needed to be rephrased to make them suitable for multiple measurements per day. From February to March 2020, the study protocol was tested via a pilot study including five Belgian participants. The original version of the EMA protocol was developed in English and then translated into Dutch and Slovenian.

Based on our triggering protocol, a semi-random sampling scheme was developed [35]. The EMAs were triggered 30 minutes into their working day, approximately every 90 minutes during the day, and during the participants’ evening routine (approximately between 8 pm and 9 pm). Both the triggering of the first EMA of the day and the evening EMA were personalised during participant briefing, being set to their preferred time. A reminder was sent after 15 minutes if the EMA got no response from the participant. They could answer the EMAs up to 90 minutes after the original trigger before a new EMA appeared. This study aimed to assess experiences as closely as possible to their actual occurrence to reduce the risk of retrospective recall bias. This was possible by taking several measurements per day via EMAs [34].

### Impact of the Covid-19 pandemic

Our data collection was scheduled to start right after the pilot study. However, due to the outbreak of the Covid-19 pandemic, it had to be postponed from March 2020 until a slow start in October 2020, finalising it in June 2021.

Initially, our STRAW smartphone application and our data collection procedure only allowed participation during work at the participants’ office. However, due to the ongoing delay of data collection, we adapted and improved our approach to also collect data during work at home and other locations outside of the office. The flexibility of the work location during participation allowed a more inclusive data collection procedure, which was then selected as a co-variable in the analysis stage. Additionally, using the participants’ own smartphones as the main data collection tool proved to be suitable in such circumstances. These protocol adaptations and using a self-developed smartphone application allowing ad-hoc changes enriched our final dataset in qualitative and quantitative aspects, making it much more suitable for the increasingly common culture of working remotely in academia.

### Measures

#### Work-related stressors

Based on the Job Demand-Control model [9] and the Job Content Questionnaire [36], job demands, job control, and the demand/control ratio (i.e., job strain) were measured with the five job demand items (e.g., *“My job required working very hard”*) and the nine job control items (e.g., *“My job allowed me to make a lot of decisions on my own”*). The reliability and validity of the Dutch and Slovenian versions of the questionnaire were confirmed by previous studies [37, 38]. Two items from each subscale were randomly selected by the STRAW application for each EMA and were asked repeatedly during the participants’ working hours. Participants answered on a 4-point Likert scale ranging from *“I strongly disagree”* (1) to *“I strongly agree”* (4). These items followed the introduction: *“Since you started working today / since the last questionnaire”*.

#### Work engagement

Work engagement, as defined by Schaufeli and Bakker [12] and as suggested by the Utrecht Work Engagement Scale [12], includes three subscales (i.e., vigour, dedication, and absorption). However, in this paper, it will be considered as one complete concept. The questionnaire was originally developed in Dutch and the reliability and validity were confirmed by Schaufeli and Bakker [12]. The reliability and validity of the Slovenian version were confirmed by previous research [39]. Two out of five vigour items (e.g., *“At my work*, *I feel bursting with energy”*), two out of five dedication items (e.g., *“I am enthusiastic about my job”*), and two out of six absorption items (e.g., *“Time flies when I’m working”*) were randomly selected by the STRAW application for each EMA and were asked once a day in the evening. Participants answered on a 5-point Likert scale ranging from *“Not at all”* (1) to *“All the time”* (5). These items followed the introduction: *“Referring to your whole working day”*.

#### Recovery experiences

Psychological detachment and relaxation, two subscales of the Recovery Experience Questionnaire [17] were included in the EMAs. Previous research confirmed the reliability and validity of the Dutch and Slovenian versions of the questionnaire [40, 41]. Two out of four psychological detachment items (e.g., *“I forget about work”*) and two out of four relaxation items (e.g., *“I kick back and relax”*) were randomly selected by the STRAW application for each EMA and were asked once a day in the evening. Participants answered on a 5-point Likert scale ranging from *“I strongly disagree”* (1) to *“I strongly agree”* (5). These items followed the introduction: *“Since you stopped working today”*.

#### Additional variables

Age, gender, country, and job category were asked at baseline by means of an online LimeSurvey questionnaire. Job category was measured with an open question. Work location was asked once a day in the evening. Participants answered the question “*Where did you do your work*?*”* with one of the following options: *“At the office”*, *“At home”*, *“I moved from between the office and home”*, or *“Other”*.

### Analysis

#### Variables

All subscales were averaged over two items per EMA. Job demands, job control, and job strain were included as independent variables. For the data analysis, daily means of both job demands and job control were calculated since they were measured several times a day. The demand/control ratio (i.e., job strain) was calculated by dividing the daily mean of job demands by the daily mean of job control. A higher ratio score implied a higher job strain. Work engagement was included as a dependent variable. The daily means of the subscales vigour, dedication, and absorption were calculated. Afterwards, the daily mean of the total work engagement score was calculated. The daily mean for both subscales psychological detachment and relaxation was calculated. They were included first for main effect testing and then as interaction terms. Time-fixed covariates included in the analysis were age (in years), gender, country (Belgium or Slovenia), and job category (administrative and technical staff, researchers without a PhD, or researchers with a PhD). One time-varying covariate included was work location. Work location was categorised as either “*At home*” or “*Non-home*”. Days, when participants did not work exclusively at home (e.g., when they worked partly at home, worked at their office, or worked at a third location), were assigned to the latter category.

#### Statistical analysis

The initial dataset included 57 participants, of which 30 participated in Belgium and 27 in Slovenia. One participant decided to withdraw participation after completing the LimeSurvey questionnaire due to a lack of time for further participation. Another person participated throughout the main data collection period but did not complete the LimeSurvey questionnaire. Consequently, these two participants were excluded from the final dataset and the data of 55 participants were analysed. All 55 participants completed the LimeSurvey questionnaire and at least 15 workdays of EMA data collection. No participant dropped out between briefing and debriefing. Participant adherence was high including a total of 6639 initiated EMAs. 81.0% were completed EMAs, 14.8% were short indicators such as *“Finished the working day”*, and 4.2% were actual incomplete EMAs [42].

We included two levels of clustered data: level 1 being repeated assessments per day, and nested within participants being level 2. We tested linear regressions between day-to-day job demands, job control, and job strain as independent variables and work engagement as a dependent variable using generalised linear mixed models. We focused on fixed-effect model testing, using repeated measures within each participant as their own control. We applied random-intercept modelling instead of random-slope modelling. First, we did not aim to model any changes during the data collection period. Second, we did not assume that the relations between our work stress exposures and outcomes would be different between participants. Third, a random-intercept model is more robust for our sample size of 55 participants. Histograms and QQ plots (showing the distribution of residual terms) were used to choose our modelling approach, allowing us to visually inspect the variables and to check the assumption of normality and homoscedasticity, in which residual terms were plotted against model-predicted values.

Based on previous research, recovery from work can be considered as an intermediate step between work stress experiences and work engagement. Accordingly, work stress during the day and recovery experiences during the evening of that day (*t1*) were matched with work engagement during the following day (*t2*). To consider a possible weekend effect of recovery on the relationship between Friday’s stress and recovery and Monday’s engagement, these data points were excluded from the data analysis. Due to these exclusions, our number of item measurements decreased from 3683 to 2710. These 2710 item measurements included work-related stressors, work engagement, and recovery experiences originating from the completed EMAs. We had 4.8% missing item measurements (59 missing item measurements of work engagement, psychological detachment, and relaxation, i.e., 177 in total, and no missing item measurements of work-related stressors).

Model I shows the confounder effect testing with selected covariates based on comparative literature. We included our independent variables for model II, dividing it into two sub-models–e.g., IIa for job demands and job control treated separately and IIb for job strain–to avoid multicollinearity. We applied this approach of sub-models for the rest of the analysis process. In models IIIa–IIId we focused on recovery experiences for main effect testing on work engagement, once psychological detachment and once relaxation. In models IVa–IVd we included recovery experiences as interaction terms, once psychological detachment and once relaxation.

Analyses were performed using R (version 4.1.0), RStudio (version 1.4.1717), and SPSS (version 27) with statistical significance determined at p < 0.05 [43].

#### Sensitivity analysis

First, we tested our results for a time effect on day-level to test if an increasing or decreasing trend in work engagement over 15 days of data collection could be observed. By means of adding the time variable *“days passed”* to the interaction models (models IVa–IVd), we checked for some sort of learning effect over time since throughout their data collection our participants started to get used to the EMA content. Second, by means of adding the time variable *“days of the week”* to the interaction models (models IVa–IVd), we tested for differences in work engagement between Monday and the rest of the work week. We applied this analysis to see if the weekend had a prolonged effect on participants’ perception of work engagement due to the recovery experiences during the weekend.

### Ethical considerations

Ethical clearance for the STRAW project was received from the commission of Medical Ethics of the Ghent University Hospital, Belgium (No. EC/2019/1091) and the Ethics Committee of the Faculty of Arts at the University of Ljubljana, Slovenia (No. 168–2019).

Before participating, all participants signed a written informed consent. At the end of their participation, they received a personalised feedback report based on their own study results. As a thank-you for their participation, Belgian participants also received a 30 Euro voucher. However, since providing incentives to participants in Slovenia is legally difficult, Slovenian participants did not receive such a monetary incentive. Since it was a modest monetary incentive, as mentioned in the protocol paper [32], the potential impact on the comparability of both samples was expected to be limited.

## Results

### Descriptive results

The descriptive statistics of the study sample are shown in Table 1. The participants’ average age was 34.2 years (SD = 9.7 years) within a range of 24 to 62 years old. As initially planned, the sample was approximately balanced in terms of gender (29 men vs 26 women) and country (26 in Slovenia and 29 in Belgium). About half of the participants were researchers without a PhD (47%). The other half were administrative and technical staff (27%) or researchers with a PhD (26%). All results of the time-varying variables in Table 1 are representative of the whole study sample across the complete data collection period. Little over half of the work by our participants was done exclusively at home (55%), as opposed to working partially at home, working at their office, or working at a third location (45%). Since higher scores indicate a better recovery experience, we saw that participants reported on average lower psychological detachment (3.2, SD = 1.0) compared to relaxation (3.6, SD = 0.9).

**Table 1:** Descriptive statistics of the study sample (N = 55). [number of item measurements = 2710; SD = standard deviation].

**Time-fixed variables**			**Mean (SD)**	**N (%)**
Demographic data	Age *(in years)*		34.2 (9.7)	
	Gender	Male		29 (53)
		Female		26 (47)
	Country	Slovenia		26 (47)
		Belgium		29 (53)
Job category		Admin and technical staff		15 (27)
		Researchers without a PhD		26 (47)
		Researchers with a PhD		14 (26)
**Time-varying variables**			**Mean (SD)**	**N (%)**
Work location ^t1^		Non-home ^a^		311 (45)
		At home		381 (55)
Job demands ^t1^		[Likert scale: 1 − 4]	2.2 (0.5)	
Job control ^t1^		[Likert scale: 1 − 4]	2.8 (0.4)	
Job strain ^t1^		Demand/control ratio	0.8 (0.2)	
Psychological detachment ^t1^		[Likert scale: 1 − 5]	3.2 (1.0)	
Relaxation ^t1^		[Likert scale: 1 − 5]	3.6 (0.9)	
Work engagement ^t2^		[Likert scale: 1 − 5]	3.3 (0.7)	

^a^ Non-home = Participants did not work exclusively at home on the questioned day. They either worked partially at home, worked at their office, or worked at a third location.

^t2^ Work engagement of the following day after t1 measurements.

### Inferential results

The Intraclass Correlation Coefficient (ICC) was calculated to obtain the proportion of variance in work engagement explained by the clustering structure of the study sample. The ICC ranging from 0 (clustering provides no information) to 1 (substantial variability between clusters) was 0.38, meaning that approximately 62% of the variance in work engagement can be explained by within-participant variability.

**Fig 1:**
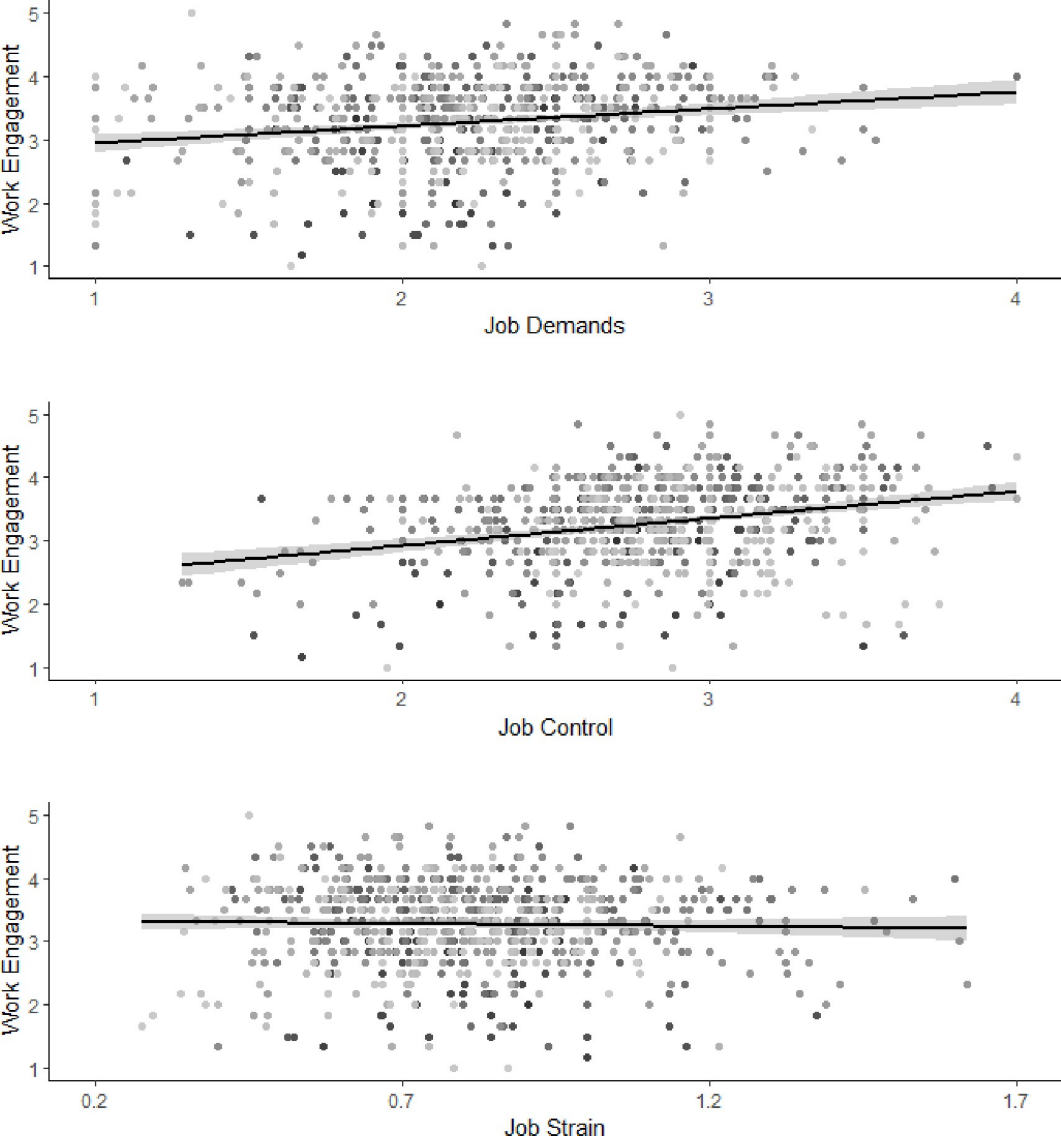
Crude associations of job demands, job control, and job strain with work engagement. For job demands and job control, items were answered on a 4-point Likert scale ranging from “I strongly disagree” (1) to “I strongly agree” (4). The demand/control ratio was calculated by dividing the daily means of job demands by the daily means of job control. For work engagement, items were answered on a 5-point Likert scale ranging from “Not at all” (1) to “All the time” (5).

The crude associations of job demands, job control, and job strain with work engagement are shown in Fig 1. The results of the random-intercept models are shown in Tables 2 and 3. Table 2 includes model I, including only the covariates, and model II, including the covariates and independent variables. The models with psychological detachment and relaxation, both as main and interaction effects, are presented in Table 3.

**Table 2:** Random-intercept models of the associations between day-to-day job demands ^t1^, job control ^t1^, job strain ^t1^, and work engagement ^t2^. [N = 55; number of item measurements = 2710; CI = confidence interval].

	Fixed-effect regression coefficient (95% CI)		
	Model I	Model II	
		IIa	IIb
**Time-fixed variables**			
**Age**	0.02 (0.00;0.03)	0.02 (0.00;0.03)	0.02 (0.00;0.03)
**Gender:** Female	0.21 (−0.03;0.45)	0.19 (−0.04;0.42)	0.20 (−0.03;0.44)
**Country:** Belgium	0.19 (−0.06;0.44)	0.13 (−0.11;0.37)	0.15 (−0.10;0.39)
**Job category:** ^a^	0.18 (−0.21;0.57)	0.15 (−0.22;0.53)	0.18 (−0.21;0.56)
Researchers without a PhD	0.27 (−0.07;0.61)	0.23 (−0.10;0.56)	0.30 (−0.04;0.63)
Researchers with a PhD			
**Time-varying variables**			
**Work location:** ^b^ At home	−0.05 (−0.17;0.08)	−0.04 (−0.16;0.08)	−0.05 (−0.17;0.07)
Job demands		0.01 (−0.13;0.15)	
Job control		**0.28 (0.13;0.43)** ***	
Job strain *(demand/control ratio)*			**−0.32 (−0.64;0.00)** *

^a^ ref. Admin and technical staff

^b^ ref. Non-home: Participants did not work exclusively at home on the questioned day. They either worked partially at home, worked at their office, or worked at a third location.

^t2^ Work engagement of the following day after t1 measurements.

* p<0.05

** p<0.01

*** p<0.001

**Table 3:** Random-intercept models of the associations between day-to-day job demands ^t1^, job control ^t1^, job strain ^t1^, psychological detachment ^t1^, relaxation ^t1^, and work engagement ^t2^. [N = 55; number of item measurements = 2710; CI = confidence interval].

	Fixed-effect regression coefficient (95% CI)								
	Model III		Model IV		Model III		Model IV		
	IIIa	IIIb	IVa	IVb	IIIc	IIId	IVc	IVd
**Time-fixed variables**									
**Age**	0.02 (0.00;0.03)	0.02 (0.00;0.03)	0.02 (0.00;0.03)	0.02 (0.00;0.03)	0.01 (0.00;0.03)	0.02 (0.00;0.03)	0.01 (0.00;0.03)	0.02 (0.00;0.03)
**Gender:** Female	0.19 (−0.04;0.42)	0.20 (−0.04;0.44)	0.18 (−0.05;0.40)	0.18 (−0.05;0.42)	0.18 (−0.05;0.41)	0.19 (−0.05;0.43)	0.18 (−0.05;0.41)	0.19 (−0.05;0.42)
**Country:** Belgium	0.13 (−0.10;0.37)	0.15 (−0.10;0.39)	0.14 (−0.10;0.38)	0.15 (−0.09;0.39)	0.16 (−0.08;0.40)	0.17 (−0.07;0.42)	0.16 (−0.08;0.40)	0.17 (−0.07;0.42)
**Job category:** ^a^	0.15 (−0.22;0.53)	0.18 (−0.20;0.57)	0.15 (−0.22;0.52)	0.17 (−0.21;0.55)	0.18 (−0.20;0.55)	0.20 (−0.18;0.58)	0.17 (−0.20;0.55)	0.20 (−0.19;0.58)
Researchers without a PhD								
Researchers with a PhD	0.23 (−0.10;0.56)	0.30 (−0.04;0.64)	0.23 (−0.10;0.56)	0.29 (−0.04;0.62)	0.24 (−0.09;0.57)	0.30 (−0.03;0.64)	0.23 (−0.10;0.56)	0.29 (−0.04;0.62)
**Time-varying variables**									
**Work location:** ^b^ At home	−0.03 (−0.15;0.09)	−0.04 (−0.16;0.08)	−0.04 (−0.16;0.08)	−0.05 (−0.17;0.08)	−0.04 (−0.16;0.08)	−0.05 (−0.17;0.07)	−0.04 (−0.16;0.08)	−0.05 (−0.17;0.08)
Job demands	0.02 (−0.13;0.16)		0.25 (−0.10;0.60)		−0.01 (−0.15;0.14)		0.10 (−0.33;0.52)	
Job control	**0.28 (0.13;0.43)** ***		0.26 (−0.17;0.68)		**0.28 (0.12;0.43)** ***		0.30 (−0.22;0.83)	
Job strain *(demand/control ratio)*		−0.32 (−0.64;0.01)		0.18 (−0.63;0.99)		**−0.35 (−0.67;−0.03)** *		−0.07 (−0.95;0.81)
Detachment	0.01 (−0.05;0.07)	0.00 (−0.06;0.06)	0.15 (−0.25;0.56)	0.13 (−0.07;0.33)				
Relaxation					−0.06 (−0.13;0.00)	**−0.08 (−0.14;−0.14)** *	0.02 (−0.48;0.52)	−0.01 (−0.22;0.20)
Demands by detachment			−0.08 (−0.18;0.03)					
Control by detachment			0.01 (−0.11;0.13)					
Job strain by detachment				−0.16 (−0.40;0.08)				
Demands by relaxation							−0.03 (−0.14;0.08)	
Control by relaxation							−0.01 (−0.14;0.13)	
Job strain by relaxation								−0.08 (−0.32;0.16)

^a^ ref. Admin and technical staff

^b^ ref. Non-home: Participants did not work exclusively at home on the questioned day. They either worked partially at home, worked at their office, or worked at a third location.

^t2^ Work engagement of the following day after t1 measurements.

* p<0.05

** p<0.01

*** p<0.001

In Table 2, model I shows that none of the covariates were significant, which remained as such throughout all models. As further presented in model II, a positive association was found between job control and work engagement (model IIa: β = 0.28, p < 0.001). Additionally, job strain was negatively associated with work engagement (model IIb: β = −0.32, p = 0.05). Additional analysis with autoregressive modelling was checked (work engagement as a predictor of next-day work engagement), which resulted in similar findings (results not shown).

Table 3 shows that job control remains positively associated with work engagement even when psychological detachment and relaxation were added to the models for main effect testing (model IIIa and model IIIc: β = 0.28, p < 0.001). In model IIId, job strain and relaxation show a negative main effect on work engagement (model IIId, job strain: β = −0.35, p = 0.03; relaxation: β = −0.08, p = 0.03). Neither psychological detachment nor relaxation were significant interaction terms in the relation between work stress experiences and work engagement.

### Sensitivity analysis

The sensitivity of the results shown in the interaction models (models IVa–IVd) was tested in two ways. First, the time effect on day-level over 15 days of data collection was added. Second, we checked if there were differences in work engagement between Monday and the other days of the work week. No significant effects were found, neither for the time effect on day-level (β = 0.01, p = 0.18) nor for differences between Monday and other workdays (model IVa: β = 0.089, p = 0.21; β = 0.10, p = 0.13; and β = 0.11, p = 0.13) (model IVb: β = 0.08, p = 0.23; β = 0.09, p = 0.19; and β = 0.10, p = 0.16) (model IVc: β = 0.10, p = 0.16; β = 0.10, p = 0.14; and β = 0.11, p = 0.15) (model IVd: β = 0.09, p = 0.21; β = 0.09, p = 0.21; and β = 0.10, p = 0.18).

## Discussion

This study researched the relationships between day-to-day job demands, job control, job strain, and next-day work engagement. Furthermore, the influence of two recovery experiences (i.e., psychological detachment and relaxation) on the next-day work engagement and the relationship between the stress exposures and work engagement were explored. This is the first time an EMA study, based on a self-developed smartphone application, was conducted to look into day-to-day stress experiences among office workers in academia. An indicative study as described in this paper aims to provide novel insights into such EMA-based research stimulating further studies looking into larger samples and other populations.

A significant positive association was found between job control and next-day work engagement, showing that higher job control among academics was associated with higher work engagement the next day. This is in line with the findings of previous studies among academic staff [10, 11, 44]. Additionally, in our study, job control remained positively associated with next-day work engagement even when the two recovery experiences were added to the models.

Furthermore, job strain was significantly negatively related to next-day work engagement, meaning that higher job strain coincides with lower work engagement the next day. Previous research confirms our results, showing that work stress has been associated with lower work engagement [25, 26].

Interestingly, we did not find a significant association between job demands and next-day work engagement. Also, previous literature showed inconsistent findings on the influence of job demands on work engagement. Some found no relationship [8, 45] confirming our results, while others found a weak relationship between job demands and both vigour and dedication [44]. Based on previous research, the association between job demands and dedication seemed to depend on the amount of organisational support. When there was a high amount of organisational support, academic staff was more dedicated, independent of the level of experienced job demands [44].

Relaxation as a main effect was significantly related to next-day work engagement, showing that higher relaxation after the working day was associated with a lower next-day work engagement. This is an interesting result, which one could interpret that a relaxing evening might impact one’s motivation to return to the office or one’s dedication and engagement to perform at work the next day. Our result is contrasting with previous research suggesting that work engagement is higher when workers could recover well from work during the previous evening [28]. However, no interaction effect of relaxation on the relationship between work stress and next-day work engagement was found.

Bennett et al. [21] showed a significant positive association between psychological detachment and vigour. However, no significant associations were found in this study between psychological detachment and next-day work engagement, neither for psychological detachment as a main effect nor as an interaction effect with stress experiences.

None of the co-variates, i.e., age, gender, country, job category, and work location, had a significant effect on work engagement.

### Strengths and limitations

The main strength of this study is the comprehensive data collection procedure based on a self-developed EMA protocol embedded in our STRAW smartphone application [33]. Due to this data collection procedure, we were provided with a large dataset including 2710 item measurements of 55 office workers–a small sample size compared to cohort studies. However, as a preparatory step for this paper, a systematic review was conducted including studies aiming to obtain repeatedly/continuously collected data on stress predictors and outcomes via EMAs or similar methods in day-to-day and real-world work environments [46]. In this systematic review, the sample sizes ranged from 14 to 304 participants. However, compared to traditional stress research, data collection in EMA studies is not only done once or twice per participant. The studies included in the systematic review collected data between one and 182 days with a sampling frequency per participant between once per week and once every 45 minutes during working hours. Therefore, this study’s data collection procedure is typical for EMA studies and makes up for its number of participants with a highly repeated measurement scheme per participant across 15 working days, revealing not only between- but also within-participant data. Moreover, despite the data collection procedure being highly demanding, participant adherence was high without drop-outs between briefing and debriefing and only 4.8% missing item measurements.

The main limitation is the usage of convenience sampling, potentially introducing selection bias. Therefore, it is relevant to mention that we most likely included workers with not only an intrinsic interest in the topic of work stress but also the capacity to participate in the study. Second, in the present study, there might be limited external validity for other academic and non-academic office jobs. Third, to limit the burden on our participants, no data was collected during weekends. Therefore, we could not investigate the recovery experienced during weekends. Consequently, data on work-related stressors and recovery experiences gathered on Fridays and data on work engagement on Mondays were excluded from the analysis. However, the time sensitivity analysis showed that there was no difference in work engagement between Mondays and any other workdays.

## Conclusions

This study confirms some of the previously published results on day-to-day work-related stressors, work engagement, and recovery experiences. First, higher job control was associated with higher work engagement the next day. Second, increased job strain predicted lower next-day work engagement.

However, no associations were found between job demands and work engagement the next day, which is in line with conflicting previous results showing either no or only weak associations. Interestingly, higher relaxation after the working day was associated with a lower next-day work engagement and no significant relation between psychological detachment and next-day work engagement was found. Consequently, further research on the associations between recovery experiences and work engagement is needed.

Based on this study, approximately 62% of the variance in work engagement can be explained at the within-participant level, showing the relevance of investigating occupational stress and its consequences on a day-to-day level.

## Supporting information

S1 Dataset (CSV)

S2 Dataset (SAV)
